# The Smithsonian Institution

**Published:** 1854-10

**Authors:** 


*The Smithsonian Institution.*—We have just laid aside, after perusal, a report of a committee of the Board of Regents of the Smithsonian Institution, relative to the propriety of making any changes in the government of that establishment.

The medical profession has an interest in the success and progress of this national scientific school, at least as deep as any other class in community. When we reflect upon its liberal endowment, and the freedom from restraint as to direction which marked the original bequest, it seems not too much to hope that from it may be derived much of benefit to the profession at large. The promise was a fair one, and the Board of Regents have from time to time indicated an intelligent appreciation of the objects of their charge.

But it seems that a curse hangs upon all government schools in this country. The pages of this journal have heretofore testified to its opinion of the workings of a system of government patronage which is unsuited to the times, the institutions of the country, or the people who inhabit it. If we are to find in the Smithsonian Institution another corroboration of our previous ideas, no one can feel a deeper regret at the unfortunate result than those who felt compelled to predict it. When our great country became the recipient of the bounty of an illegitimate scion of English nobility, and stepped in between the lawful heirs and their just rights, there was little to be hoped from so ominous a beginning.

For some time past there have been leakings out of difficulties among the professors of this school, of heart-burnings and jealousies, of contradictory plans and selfish designs. So near as we can ascertain, two parties now exist among them. Our sources of information are not sufficiently abundant to enable us to give a very lucid account of the controversy, but we know enough to render it evident that the medical interests of the country are not those the most nearly cared for, or the least likely to suffer from a deprivation of the little public sustenance it now receives.

Of the two parties we mention, one wishes to establish a huge library—another British Musuem—in which shall be gathered all books from all lands, to be nicely shelved in the alcoves of a stately building, for the benefit of—a dozen or two of bookworms. The great mass of the people, the men of science and research scattered throughout the country, can, by making an expensive pilgrimage of some hundreds or thousands of miles, reach this repertory of knowledge, and draw from it the information they desire. It would be a bookworm's paradise, but, alas! as hopeless and unattainable to the seedy-coated individuals who should profit by it most, as any other paradise!

The other party, (and we are rejoiced to see that the Board of Regents sustain them,) would rather diffuse than concentrate knowledge. They would allow the library to be a work of years, and, in the meantime, would devote the larger share of the income to original research in the arts and sciences. They would extend their observations to climate, soil, agriculture, geography, geology and chemistry; and fill up, so far as possible, the wide gaps in our knowledge of them. Calling to their aid the enthusiasm of the scattered amateurs of the nation, they would make the Smithsonian truly a national institution.

The reader may by this time inquire what special interest has the medical profession in this controversy? How shall it be benefited by the adoption of either plan?

If, then, we can show that the profession has a deep and personal stake in this matter, it will be a sufficient apology for obtruding upon our readers a subject apparently so foreign to their interests.

When the Institution commenced its operations, a department of meteorology was established, and placed under the charge of Mr. Lorin Blodget, a gentleman of high scientific attainment, and warm enthusiasm in his specialty. Two projects were offered. Some of Mr. Blodget's associates wished to establish a few elaborate and expensive observatories in different sections of the country, to be placed under the charge of paid observers. To this it was objected that the plan, while expensive, would furnish but a barren result; that proper statistics could not be thus collected in a country like this, extended, and presenting, not unfrequently, in narrow limits, great varieties in climate and disease. An extended series of observations was needed, which, occupying numerous points in all sections, should express, when gathered and reduced to form, the totality of the meteorology of the country.

Mr. Blodget, calculating upon the zeal of amateur observers, offered to establish with their aid a series of observations which should furnish all the data necessary. To this end, the plan being adopted, he distributed a large number of instruments to scientific men in the most advantageous localities, and in a few months of hard labor on his part, seconded by the hearty coöperation of unpaid, but reliable assistants, he had established some two hundred different stations, at which daily records were made of the state of the barometer, thermometer and hygrometer, and of the phenomena of winds, clouds, rain and storms. The result proved the wisdom of the plan. The statistics accumulated were reliable and ample, and the department, after one or two years of observation, had at a trifling expense secured the possession of the most valuable mass of meteorological statistics ever accumulated in any country.

To classify these must necessarily have involved a vast amount of labor, but the value of them was such as to richly repay its outlay. Much was expected from them. Closely associated as they are with the epidemics of the country, and occupying an unexplained relation to their causation and cure, the profession readily recognized their importance, and were willing to accord to them a careful consideration; while they acknowledged a deep obligation to Mr. Blodget for his energy and zeal.

But thus far they have heard nothing from these results. They are a buried treasure to which no access can be had. Not only does the profession feel aggrieved, but individual observers, conscious that their own records possessed value only as a part of a grand and united whole, have grown dissatisfied and careless, and it is now evident that the whole system will soon fall through for want of proper care at the Smithsonian Institution.

Mr. Blodget is of course not the responsible party. His own fame and credit demand that his labors should be published, and that the medical world should have the benefit of them. We cannot, therefore, suppose that he stands in the way of publication, for up to the present time there has been no evidence of want of zeal or energy on his part.

Why they are not published we cannot explain, further than that the cause lies somewhere in this controversy as to the true objects of the Institution. In this struggle between centralism and diffusion, the profession of medicine is left like a suitor in chancery, prayerfully hoping that some day or other the matter may be decided, and its just claims acknowledged. In the meantime other and less worthy researches have been published at the expense of the fund. Prof. Espy, the "storm-king," a government pet of many years standing, received last year $2000—for what Heaven only knows! If he cannot offer a satisfactory theory of storms, he is at least a proficient in "raising the wind!" And this is done while the precious results of the observations of 200 meteoric stations remain unpublished for want of funds! H.

